# The Clinical Assessment of Eccentric and Concentric Stepping Kinetics has Utility in Older African American Men with Knee Arthritis

**DOI:** 10.2174/0118746098312415241112103614

**Published:** 2024-12-02

**Authors:** Tomas I. Gonzales, Bryant A. Seamon, Katie L. Boncella, Haniel J. Hernandez, Valerie McIntosh, Marc R. Blackman, Michael O. Harris-Love

**Affiliations:** 1 Muscle Morphology, Mechanics, and Performance Laboratory, Rocky Mountain Regional Veterans Affairs Medical Center, Aurora, CO 80045-7211, USA;; 2 Division of Physical Therapy, Department of Rehabilitation Sciences, Medical University of South Carolina, Charleston, South Carolina, SC 29425, USA;; 3 Department of Medicine, Biochemistry & Molecular Medicine, The George Washington University, Washington, DC 20007, USA;; 4 Department of Medicine and Rehabilitation Medicine, Georgetown University School of Medicine, Washington, DC 20007, USA;; 5 Research Service, Veterans Affairs Medical Center, Washington, DC 20422, USA;; 6 Department of Physical Medicine & Rehabilitation, The University of Colorado Anschutz Medical Center, Aurora, CO 80045, USA;; 7 Eastern Colorado Geriatric Research Education and Clinical Center, Rocky Mountain Regional VA Medical Center, Aurora, Colorado, CO 80045, USA

**Keywords:** Osteoarthritis, gait, stair navigation, kinetics, knee, eccentric muscle actions, concentric muscle actions, coordination

## Abstract

**Introduction:**

Stair navigation is physically demanding for individuals with knee osteoarthritis and may result in movement asymmetries that can be quantified using kinetic analysis and force-time parameters. Thus, the purpose of this cross-sectional study was to determine if kinetic force-time parameter asymmetries are present in individuals with knee osteoarthritis and associated with functional outcomes.

**Methods:**

Forty-six older male veterans (61.6 ± 5.6 years) participated. More and less involved legs were defined using the Kellgren and Lawrence (KL) scale and self-reported pain. Kinetics were measured with the Neurocom^®^ Step Up and Over test and quantified with the lift-up index, impact index, movement time, and stair-stepping smoothness. Smoothness was calculated from the level of intermittency in acceleration and deceleration during the concentric and eccentric test movements.

**Results:**

Smoothness was the only force-time parameter to demonstrate an asymmetry. Greater smoothness values were observed for the less-involved leg (*p*<0.001, 95% CI: 1.22 to 3.64, *d*=1.17) and were positively associated with gait speed (more-involved: *r*=0.47, *p*<0.01; less-involved: *r*=0.53, *p*<0.01), Knee Injury and Osteoarthritis Outcome Score (KOOS) Symptom (more-involved: *r*=0.31, *p*<0.05; less-involved: *r*=0.39, *p*<0.01) and activities of daily living (more-involved: *r*=0.32, *p*<0.05; less-involved: *r*=0.39, *p*<0.05) subscales, and isokinetic knee extension strength (more-involved: *r*=0.31, *p*<0.05; less-involved: *r*=0.42, *p*<0.01).

**Conclusion:**

Stair-stepping smoothness was diminished in the more involved leg and was associated with worse gait speed, patient-reported outcomes, and knee strength. This observation may reflect compromised motor control associated with decreased strength and greater disease severity in the more-involved leg.

## 1 INTRODUCTION

Stair navigation presents a significant challenge for people with knee osteoarthritis. Peak knee joint moments during stair descent are typically much greater than during stair ascent or level-ground walking [1, 2]. This increased stress can be problematic for people with knee osteoarthritis, exacerbating pain and promoting the development of compensatory movement patterns. These can manifest as alterations in gait and posture that cause symmetrical and reciprocal tasks to appear asymmetrical. Over time, this asymmetry can result in increased stress on the less-involved leg, potentially predisposing individuals to a wide range of chronic orthope-dic conditions and a decline in functional independence [3-7]. Given the potential for functional decline, therapeutic interventions are crucial for managing knee osteoarthritis by improving muscle strength, restoring movement symmetry, and reducing the risk of further injury and disability [3, 4, 8].

The ways in which movement asymmetry manifests in people with knee osteoarthritis can vary depending on the activity. During antalgic gait, the asymmetry can be characterized by increased pain with weight-bearing and a shortened stance time on the more involved leg [7]. This compensatory strategy is often accompanied by decreased knee flexion excursion during weight-bearing, further exacerbating mechanical stress on the knee joint [9]. During stair navigation, asymmetrical movement patterns similar to gait can be observed, likely due to higher knee joint moments than level-ground walking [1, 2, 10]. These can inadvertently increase fall risk during stair navigation, particularly during stair descent [10]. Obesity can exacerbate these challenges by increasing knee pain and ankle joint loads during stair descent, compromising stability and increasing the likelihood of falls [11].

Clinical assessments for stair navigation are limited, thus complicating the quantitative and longitudinal evaluation of this task [12]. This can make it difficult for clinicians to tailor interventions effectively and track progress objectively. The current gold-standard approach for quantifying stair navigation performance is functional movement analysis, which can measure force-time parameters during the eccentric and concentric phases of the stepping cycle [13]. These data are typically collected using instrumented force plates and 3D motion capture, but equipment costs limit clinical utility.

**Fig. (1):**
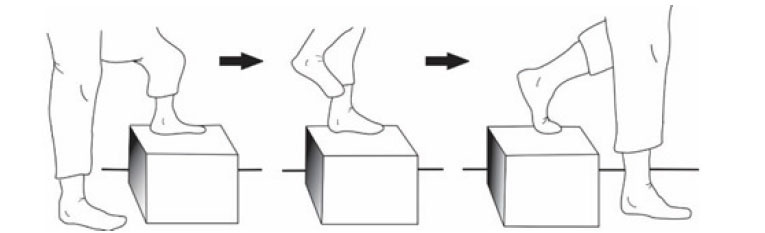
A visual depiction of the Step Up and Over Test (SUO). Participants begin in quiet standing before placing their test leg onto the box. Participants then step up and over the box using their test leg in one fluid motion. The test is ended when the patient brings the test leg down next to the non-test leg in quiet stance.

The Step Up and Over (SUO) test, which involves ascending and descending a step while on force plate platform (Fig. **1**), has been previously used to assess weight shifting in individuals with neurological deficits and lower extremity performance asymmetries in people with a history of anterior cruciate ligament repair (ACL) [14-17]. The SUO test allows clinicians to quantify movement performance during stair navigation and identify asymmetries between the more- and less-involved legs. However, the clinical utility of the SUO in characterizing the eccentric and concentric phases of stepping kinetics for people with knee osteoarthritis is unclear. This information could be beneficial in tailoring targeted interventions to improve stair navigation performance and reduce fall risk.

The purpose of this study was to determine the clinical utility of the SUO test in a sample of older African American men with knee osteoarthritis. Our primary aims were to: **1**) use the SUO test parameters to quantify step navigation performance; and **2**) determine the association of SUO test parameters and stepping kinetics asymmetries with knee strength, gait speed, self-reported activities of daily living (ADL), and self-reported disease status.

## 2 MATERIALS AND METHODS

### 2.1 Participants

Forty-six older, overweight, and obese African American male veterans (mean ± standard deviation 61.6 ± 5.6 years; 31.9 ± 6.3 BMI) with knee osteoarthritis participated in this cross-sectional study. Participants were consecutively recruited from the Rheumatology Service, Geriatrics Service, and Primary Care Medical Service at the Washington DC Veterans Affairs (VA) Medical Center. The study was approved by the Washington DC VA Medical Center Institutional Review Board (IRB) and Research & Development Service and registered with Clinicaltrials.gov (NCT020980 96). All study participants gave written informed consent in accordance with the Declaration of Helsinki and signed informed consent was obtained from all participants prior to data collection. All participants were sedentary with less than three exercise sessions per week for three consecutive months prior to participation. Exclusion criteria included any major surgical procedure within the last six months, non-ambulatory status, neurogenic weakness, and endocrine or metabolic disorders that cause excessive fatigue or muscle weakness.

### 2.2 Measures of Disease Status and Functional Performance

Knee osteoarthritis severity was evaluated according to the Kellgren and Lawrence (KL) classification criteria. KL grade classifications range from 1 (least severe) to 4 (most severe) depending on the degree of joint space narrowing, the presence of subchondral sclerosis, and osteophyte formation [18]. Participant knee radiographs were reviewed by a physician, and the more involved leg was defined as the knee with the higher KL grade. For those participants with the same KL grade for both knees, the self-reported more painful leg was considered the more involved leg [19]. The assessment of knee osteoarthritis severity (most-involved leg) among the study participants included 20 people at Grade 2, 18 people at Grade 3, and 8 people at Grade 4.

Self-reported symptoms and physical function were evaluated using the Knee Injury and Osteoarthritis Outcome Score (KOOS), which consists of five subscales: Pain, Symptoms, Activities of Daily Living (ADL), Sport and Recreation Function (Sport/ Recreation), and knee-related Quality of Life (QOL). A normalized score ranging from 100 (no symptoms) to 0 (extreme symptoms) was calculated for each KOOS subscale using established methods [20].

Gait function was measured using the 50-foot Walk Test [21]. Participants were timed while they walked a premeasured 50-foot distance (15.24 m) at a self-selected speed. Body mass and height substantially influence energy expenditure [22] and gait speed [23] in older adults; therefore, gait speeds were scaled to participant height as statures/s. Knee extensor and flexor strength were assessed *via* isokinetic dynamometry at 60°/s and 180°/s (Biodex System 4, Biodex Medical Systems, Shirley, NY) according to published protocols by the authors and other investigators [24, 25]. Briefly, the participants were tested in the seated position at 90° of hip flexion and knee flexion with knee stabilization straps appended at the shoulders, pelvis, ipsilateral thigh, and ipsilateral lower leg at the level of the attachment pad. The seat height was adjusted to ensure the alignment of the lateral femoral condyle with the axis of rotation of the dynamometer shaft. Participants completed five maximal repetitions of reciprocal isokinetic knee extension and flexion at the selected angular velocity. The testing order was randomly determined. Approximately one minute of rest was provided between the bouts of isokinetic testing. Visual feedback was provided to the participants using the torque-time curve displayed on the dynamometer computer screen, and verbal curing was used as needed to address attempted compensatory motions during testing. A familiarization session preceded all strength testing visits, and strength values were scaled to participant body weight.

### 2.3 Experimental Setup and Procedures

Step ascent and descent performance were assessed using the Neurocom^®^ SUO test (Fig. **1**). A wooden block (dimensions, 40.0 cm × 40.0 cm × 20.3 cm) was placed at the center of a long force plate (dimensions, 152.0 cm × 46.0 cm × 5.0 cm; Fz capacity, 1800 N; Fx capacity, 180 N; Fy capacity, 180 N; sampling rate, 100 Hz; maximum participant body weight, 227 kg). Foot placement at the start of the test was standardized by aligning the lateral malleolus with markings on the force platform. Participants were instructed to step up onto the block with their test leg, swing their non-test leg up and over the box marking a transition from step ascent to step descent, and then place the non-test leg onto the force platform. The test was completed when participants stepped down from the platform with their test leg and returned both feet to the force platform. The terminal position was maintained for 5 seconds to ensure complete movement trajectory data collection. Three trials were conducted with each leg consecutively for a total of six trials. Participants could self-select whether they performed the first three trials of the test with their less-involved or more-involved leg as the test leg. A physical therapist was present during testing to prevent falls, and approximately 30 seconds were allotted for rest between trials.

### 2.4 Data Acquisition and Analysis

Ground reaction force and center of pressure data were sampled at 100Hz (Neurocom^®^ Equitest). Performance variables computed by the system software were the lift-up index, impact index, and movement time. The lift-up index was defined as the ratio between the participant’s body weight and the maximum amount of force applied by the test leg during the steep ascent. The impact index was defined as the ratio between body weight and the maximum amount of force applied by the non-test leg when stepping off the block. Movement time was defined as the total time to complete the test.

**Fig. (2):**
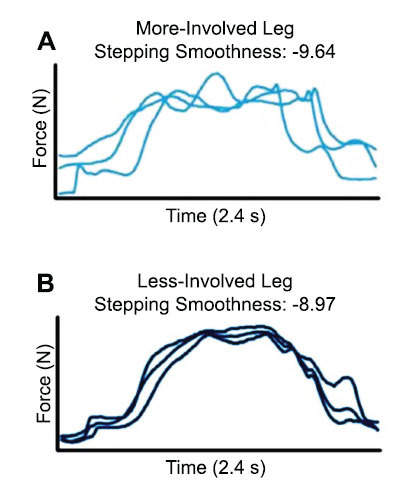
Stepping smoothness kinetics during the Step Up and Over Test. A schematic representation of stepping smoothness derived from exemplar force-time curve data during the “step up” phase of the Step Up and Over test. Each tracing represents one trial. Stepping smoothness was lower (*p* < 0.05) in the more-involved leg (**A**) compared to the less-involved leg (**B**) across the study sample.

Stair-stepping smoothness was calculated from ground reaction forces in LabVIEW (National Instruments, Austin, TX, USA). Ground reaction force data were filtered with a 2nd-order low-pass Butterworth filter (cut-off frequency of 15 Hz) and used to compute stepping smoothness (SS) according to the following equation:


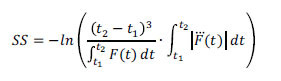


where F(t) is the force-time curve signal from the lead leg and t_1_ and t_2_ are the start (when the test leg makes initial contact with the block) and end (just prior to when the non-test leg made initial contact with the force plate) times of the test leg’s step. The variables t_1_ and t_2_ were determined using a threshold detection algorithm of ± 2 standard deviations from the baseline force signal and verified by visual inspection. These time points allowed the capture of the concentric ascent and eccentric descent of stair navigation for the test leg. Stair-stepping smoothness is dimensionless; it does not depend on the amplitude or duration of the force-time curve [26]. Therefore, the smoothness measure minimizes the impact of varied body weight and stepping duration across participants, thus facilitating data interpretation. Greater stair-stepping smoothness values have been hypothesized to reflect better lower-leg motor coordination when stepping on and off the block (Fig. **2**) [26].

To quantify asymmetries between the less-involved leg and more-involved leg, symmetry indices (SI) were computed for all gait and strength variables [27]:


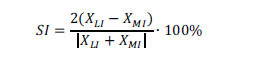


where X_LI_ is a variable for the less-involved leg and X_MI_ for the more-involved leg. A value of zero for symmetry indices indicates that there is no difference between less-involved and more-involved legs. Positive symmetry indices values indicate that the magnitude of the less-involved leg is greater than the more-involved leg, and negative values that more-involved leg is greater than less-involved leg.

### 2.5 Statistical Analysis

Statistical analyses were performed using IBM-SPSS (version 23; IBM, Armonk, New York). Descriptive statistics and visual analysis of box plots were used to assess the data distribution and variance for departures from normality. Single sample t-tests were performed for each symmetry indices parameter to determine whether mean differences between the less and more involved legs were statistically significantly different than zero. Confidence intervals of 95% (95% CI) were calculated for each symmetry index, and Cohen’s effect sizes (*d*) were calculated. Effects sizes were interpreted as 0.00-0.20=trivial, 0.20-0.50=small, 0.50-0.80=moderate, >0.80=large [28]. Correlations between KOOS subscale scores, participant characteristics, isokinetic knee extensor strength, and gait performance parameters were calculated by Pearson product-moment correlation (*r*) analysis. Pearson’s *r* was interpreted as trivial ± 0.10, small ± 0.11-0.30, moderate ± 0.31-0.50, large ± 0.51-0.70, very large ± 0.71-0.90, and nearly perfect ± 0.91-0.99 [28].

## 3 RESULTS

**Fig. (3):**
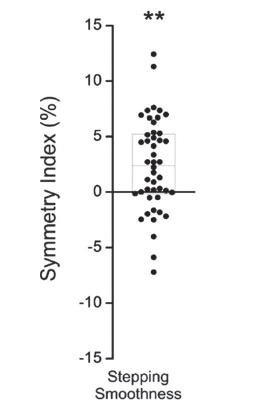
Stepping smoothness symmetry index from the Step Up and Over test. The overall symmetry index for stair-stepping smoothness was significantly different from zero (perfect symmetry) and was positive in magnitude indicating that the less-involved leg had more smooth movement compared to the more-involved leg (Single sample t-test; ** *p* < 0.01).

**Fig. (4):**
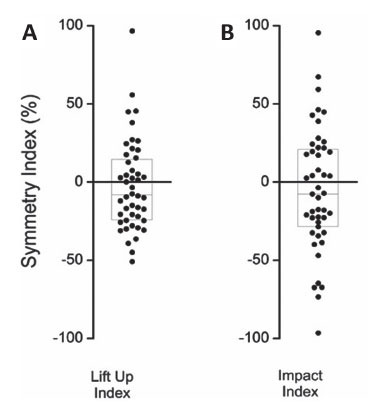
Lift-up index and impact index symmetry indices from the Step Up and Over test. The overall Lift Up Index (**A**) and Impact Index (**B**) were not significantly different from zero (perfect symmetry) (Single sample t-test; *p* > 0.05).

**Fig. (5):**
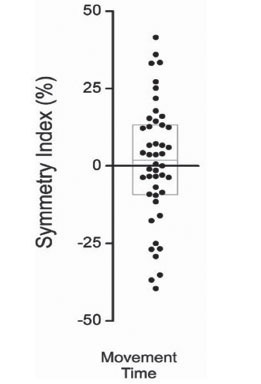
Movement time symmetry index from the Step Up and Over test. The overall Movement Time was not significantly different from zero (perfect symmetry) (Single sample t-test; *p* > 0.05).

Participant characteristics of the 46 African American male veterans are provided in Table **1**. The mean symmetry index for stair-stepping smoothness (Fig. **3**) was greater than zero (*p*<0.001, 95% CI: 1.22 to 3.64, *d*=1.17), indicating that stair-stepping kinetics were smoother when the less-involved leg was the test leg during the step-up phase of stair navigation. Differences were not observed for mean symmetry indices of the lift-up index (*p*=0.598, 95% CI: -10.69 to 6.14, *d*=-0.16) and impact index (*p*=0.393, 95% CI: -16.36 to 6.37, *d*=-0.26; Fig. **4**). Additionally, non-significant differences (Fig. **5**) were noted for movement time based on the mean symmetry index for the less and more involved legs (*p*=0.670, 95% CI: -4.39 to 6.85, *d*=0.13). When isokinetic knee extensor strength was evaluated at 180º/s, scaled strength values were greater in the less-involved leg (*p*=0.019, 95% CI: 2.15 to 19.65, *d*=0.73). Strength asymmetries were not observed in the 60º/s testing condition (*p*=0.437, 95% CI: -6.36 to 14.88, *d*=0.24). Participants reported moderate to severe ratings of knee osteoarthritis *via* the KOOS questionnaire. Moderate domains of involvement included the Pain (52.7 ± 19.3), Symptoms (54.3 ± 16.4), and ADL (54.0 ± 20.7) subscales, whereas more severe ratings included the Sports/Recreation (31.8 ± 22.5) and QOL (29.4 ± 22.5) subscales. Stair-stepping smoothness in the more and less-involved legs was positively associated with gait speed (more-involved: *r*=0.47, *p*<0.01; less-involved: *r*=0.53, *p*<0.01) and KOOS Symptom (more-involved: *r*=0.31, *p*<0.05; less-involved: *r*=0.39, *p*<0.01) and ADL (more-involved: *r*=0.32, *p*<0.05; less-involved: *r*=0.39, *p*<0.05) subscales. In addition, greater isokinetic strength (peak torque knee extension at 60º/s) was associated with smoother stair-stepping smoothness (more-involved: *r*=0.31, *p*<0.05; less-involved: *r*=0.42, *p*<0.01). The associations between the primary participant outcomes and asymmetries in stair-stepping kinetics based on smoothness measures are summarized in Table **2**.

## 4 DISCUSSION

The objectives of this study were two-fold. First, to determine if step ascent/descent movement asymmetries exist for individuals with knee osteoarthritis using kinetic and force-time parameters obtained during a stair-stepping activity. Secondly, we aimed to determine the association between asymmetrical stair-stepping performance and measures of gait speed, self-reported functional status, and knee strength. This study is among the first to report differences in stair-stepping smoothness between the more-involved and less-involved leg in adults with knee osteoarthritis.

In clinical settings, force-time parameters can be used to quantify performance during step ascent and descent during stair navigation and to assess kinetic asymmetries in people with symptomatic knee osteoarthritis. The study results suggest that force-time data, expressed as stair-stepping smoothness, are associated with asymmetry in knee osteoarthritis severity and lower extremity strength. The force-time profile of movement asymmetry during the experimental maneuver may have reflected reduced postural control during the single-stance phase of the stair-stepping task. Previous reports have focused on center-of-pressure trajectory smoothness during gait initiation [29] and reaching from a standing position [30]. Importantly, the smoothness measure during stair-stepping kinetics was directly related to habitual gait speed and is significantly associated with normalized patient-reported KOOS scores regarding knee osteoarthritis symptom severity and activities of daily living. This supports the notion that smoothness may have clinical utility, in addition to validity, for persons with knee osteoarthritis.

Surprisingly, bilateral differences were not observed across the standard SUO kinetic outcome measures. Both SUO kinetic measures reflect the net vertical ground reaction forces used during step ascent by the test leg (*i.e.*, the lift-up index) and weight acceptance of the non-test leg during step descent (*i.e.*, the impact index). Lower extremity impairments such as pain or muscle weakness in the test leg would be expected to result in higher impact index values due to a compromised ability to control the descending leg upon contact with the force platform [6]. However, significant differences in the impact index performance were not detected between the participant’s more- and less-involved legs. Previous observations from studies involving the SUO test and the impact index have been variable. Chmielewski *et al*. [31] detected significant differences in the impact index between those with ACL reconstruction and control participants, whereas Mattacola *et al*. [15] found that the impact index did not discriminate between those with and without this form of surgical repair. Additionally, a study featuring a comparison between patients with neurological disease and age-matched control participants also failed to show significant group differences in impact index values [5]. The findings in this study were consistent with previous reports concerning the general association of isokinetic strength values with movement time during the SUO test [15, 31]. Nevertheless, neither the movement time of the stair-stepping maneuver nor the lift-up index during step ascent was significantly different based on the knee osteoarthritis asymmetries of the study participants. While knee extensor and flexor strength significantly differed between the more-involved and less-involved leg, this was observed only for the relatively high-velocity 180º/s testing condition. The lack of differences in bilateral SUO kinetic measures such as the lift-up index and movement time may be partially explained by the similar levels of bilateral strength in the 60º/s testing condition given the presumed association of slower isokinetic strength values with controlled functional movements [32]. In addition, the magnitude of differences in SUO kinetic measures may have been larger in previous reports involving between-group analyses of post-surgical populations and healthy control participants [15, 31] in comparison to the within-group analyses concerning a sample with a chronic condition as presented in this work. Muscle strength continues to have relevance for the functional status of older adults with knee osteoarthritis and resistance exercise remains an important aspect of the conservative management of this musculoskeletal disorder [16, 33, 34]. The positive association between the high-velocity isokinetic strength values and stair-stepping smoothness observed in the study participants suggests that resistance exercise may benefit motor performance in people with knee osteoarthritis.

Varying workloads of resistance exercise may have been suggested to improve lower extremity motor control [35]. Modes of physical activity that involve active muscle lengthening, such as eccentric resistance exercise, may help improve lower extremity control during stair descent in people with knee osteoarthritis [36]. Deficits in the motor control of older adults have been noted during lengthening muscle actions in comparison to shortening muscle actions [37]. However, this observed difference may be decreased with strength training [37-39]. Early work concerning the potential value of therapeutic exercise for knee osteoarthritis using active muscle lengthening has been previously reported by the authors and other investigators [36, 40]. More efficient motor unit synchronization, featuring the optimal recruitment of smaller motor units, has been proposed as a possible mechanism for post-strength training improvements in lower extremity motor control [38]. Additional investigation will be needed to better understand the efficacy of strengthening exercises featuring active muscle lengthening to improve stair-stepping smoothness and other measures of motor coordination. Also, while measures of lower extremity motor control may be associated with functional task performance [41], this relationship has not been consistently observed by other investigators [42]. The relationship between motor control and functional status, along with the effects of strength training on lower extremity motor control, are affected by testing conditions and the method used to assess limb motion and task performance [39, 43]. The findings in this work concerning the association of smoothness measures with lower extremity movement asymmetries should be further examined in a variety of testing conditions involving other upright mobility tasks.

## 5 STUDY LIMITATIONS

This study had several limitations. All participants were older, obese African American men and recruited from a single U.S. VA medical center. Clinical management of knee osteoarthritis may differ in other population groups based on regional variations in care and patient demographics at non-VA facilities, which further limits the generalizability of the findings. Additionally, this study did not include a control group, which limits conclusions on how the reported findings differ from age-matched individuals without knee osteoarthritis. Furthermore, we did not randomize the lead limb that the participant used for the first three trials, limiting control over potential confounders such as limb dominance. While reasonable assumptions can be made regarding the use of the SUO test as a proxy measure of stair-stepping performance, the SUO kinetic measures should be compared to quantitative kinetic and kinematic analysis during stair navigation. Moreover, the isokinetic strength assessment was limited to the knee joint in this study. While knee extensor and flexor strength values were significantly associated with key SUO kinetic measures, important relationships involving other lower extremity muscles could be examined in the future. Despite these limitations, the SUO test appears to provide a useful method to detect and quantify asymmetries in people with symptomatic knee osteoarthritis during the common mobility task of stair-stepping. While ample research has been conducted on upper extremity smoothness during point-to-point reaching [44], additional work is needed to standardize protocols for assessing the smoothness of rhythmic lower extremity movements and gait kinetics [26]. The ability to objectively measure kinetic deviations in the more-involved and less-involved leg may aid rehabilitation efforts directed at improving weight-shifting patterns and appropriate load acceptance during the stair-stepping maneuver [45]. Comprehensive validation of the SUO test would involve 3D motion capture of people with knee osteoarthritis during stair step ascent and decent with integrated kinematic and kinetic gait analysis. However, given the limited options to characterize stepping performance in the clinic beyond measures of speed and qualitative observation, the SUO test may facilitate the clinical measurement of lower extremity kinetics and identification of asymmetries during the negotiation of a step. An additional study will also be needed to understand how resistance exercise, particularly forms of strengthening that involve lengthening muscle actions, may enhance motor control, and reduce stair-stepping asymmetries. Finally, prospective intervention studies may reveal if improved stair-stepping smoothness measures are associated with improved clinical outcomes in people with knee osteoarthritis.

## CONCLUSION

The present study applied the characterization of lower extremity movement smoothness to a dynamic stair-stepping task, and the presence of asymmetries between the more and less involved legs suggests the measures are valid for persons with knee osteoarthritis. Using a task-oriented stair-stepping paradigm, we found that the SUO test could identify and quantify asymmetrical kinetics based on stair-stepping smoothness in older adults with knee osteoarthritis. This observation may reflect compromised motor control associated with reduced strength and greater disease severity in the more involved leg.

## AUTHORS’ CONTRIBUTIONS

The authors confirm their contribution to the paper as follows: study conception and design: HH; data collection: BS, VM; analysis and interpretation of results: TG, MB; draft manuscript: KB, MHL. All authors reviewed the results and approved the final version of the manuscript.

## Figures and Tables

**Table 1:** Descriptive participant characteristics.

Age (y, mean ± SD)	61.6 ± 5.6
BMI (kg/m^2^)	31.9 ± 6.3
Gait statures	1.75 ± 0.41
Disease severity – self report	
KOOS Pain	52.7 ± 19.3
KOOS Symptom	54.3 ± 16.4
KOOS ADL	54.0 ± 20.7
KOOS Sport/Rec	31.8 ± 22.5
KOOS QOL	29.4 ± 22.5
Disease severity – radiographic	
KL Grade - Less Involved	2.0 (2.0-3.0)
KL Grade - More Involved	3.0 (2.0-3.0)

**Note: ** Continuous measures are presented mean and standard deviation, and categorical variables are presented as median and range.

**Table 2:** Association among primary participant outcomes and asymmetries in stair-stepping kinetics based on stepping smoothness.

**Participant Outcomes**	**Stair-Stepping Smoothness**	
	**Less-Involved Leg**	**More-Involved Leg**
Gait Speed	0.53**	0.47**
KOOS		
*Pain*	0.35*	0.28
*Symptom*	0.39**	0.31*
*ADL*	0.39**	0.32*
*Sport/Rec*	0.19	0.13
*QOL*	0.13	0.09
Knee Extensor Peak Torque (180º/s)		
*Less-Involved Leg*	0.38*	0.28
*More-Involved Leg*	0.41**	0.32*
Knee Flexor Peak Torque (180º/s)		
*Less-Involved Leg*	0.35*	0.28
*More-Involved Leg*	0.30	0.28
Knee Extensor Peak Torque (60º/s)		
*Less-Involved Leg*	0.42**	0.37**
*More-Involved Leg*	0.46**	0.31*
Knee Flexor Peak Torque (60º/s)		
*Less-Involved Leg*	0.40**	0.37**
*More-Involved Leg*	0.42**	0.29

**Note: ** Pearson product-moment correlation analysis. Peak torque values are scaled to body weight. *: *p*<0.05. **: *p*<0.01.

## Data Availability

The United States Department of Veterans Affairs (VA) places legal restrictions on access to veteran’s health care data, which includes both identifying data and sensitive patient information. The analytic data sets used for this study are not permitted to leave the VA firewall without a Data Use Agreement (DUA). This limitation is consistent with other studies based on VA data. However, VA data are made freely available to researchers behind the VA firewall with an approved VA study protocol.

## LIST OF ABBREVIATIONS

ACL: Anterior Cruciate Ligament
ADL: Activities of Daily Living
KL: Kellgren and Lawrence
KOOS: Knee Injury and Osteoarthritis Outcome Score
QOL: Quality of Life
SD: Standard Deviation
SUO: Step Up and Over
VA: Veterans Affairs
